# A Microfluidic Biosensor for Quantitative Detection of *Salmonella* in Traditional Chinese Medicine

**DOI:** 10.3390/bios15010010

**Published:** 2024-12-27

**Authors:** Yutong Wu, Yang Liu, Jinchen Ma, Shanxi Zhu, Xiaojun Zhao, Huawei Mou, Xuanqi Ke, Zhisheng Wu, Yifei Wang, Sheng Lin, Wuzhen Qi

**Affiliations:** 1 School of Chinese Materia Medica, Beijing University of Chinese Medicine, Beijing 102488, China; 2 School of Pharmacy, Shandong University of Traditional Chinese Medicine, Jinan 250355, China; 3 China Center for Information Industry Development, Beijing 100036, China; 4 Fangshan Hospital of Beijing University of Chinese Medicine, Beijing 102400, China; 5 Dongzhimen Hospital of Beijing University of Chinese Medicine, Beijing 100700, China

**Keywords:** microfluidics, biosensor, nanocatalysts, microbial contamination, traditional Chinese medicine

## Abstract

Microbial contamination is an important factor threatening the safety of Chinese medicine preparations, and microfluidic detection methods have demonstrated excellent advantages in the application of rapid bacterial detection. In our study, a novel optical biosensor was developed for the rapid and sensitive detection of *Salmonella* in traditional Chinese medicine on a microfluidic chip. Immune gold@platinum nanocatalysts (Au@PtNCs) were utilized for specific bacterial labeling, while magnetic nano-beads (MNBs) with a novel high-gradient magnetic field were employed for the specific capture of bacteria. The immune MNBs, immune Au@PtNCs, and bacterial samples were introduced into a novel passive microfluidic micromixer for full mixing, resulting in the formation of a double-antibody sandwich structure due to antigen–antibody immune reactions. Subsequently, the mixture flowed into the reaction cell, where the MNBs-*Salmonella*-Au@PtNCs complex was captured by the magnetic field. After washing, hydrogen peroxide-tetramethylbenzidine substrate (H_2_O_2_-TMB) was added, reacting with the Au@PtNCs peroxidase to produce a blue reaction product. This entire process was automated using a portable device, and *Salmonella* concentration was analyzed via a phone application. This simple biosensor has good specificity with a detection range of 9 × 10^1^–9 × 10^5^ CFU/mL and can detect *Salmonella* concentrations as low as 90 CFU/mL within 74 min. The average recoveries of the spiked samples ranged from 76.8% to 109.5%

## 1. Introduction

*Salmonella* is a bacterium highly prone to infecting the human body, leading to symptoms such as diarrhea, gastric cramps, food poisoning, typhoid fever, and in severe cases, even death [1,2]. According to Centers for Disease Control and Prevention (CDC) estimates, *Salmonella* is responsible for approximately 1.35 million illnesses, 26,500 hospitalizations, and 420 deaths in the United States annually [3]. In the field of traditional Chinese medicine, *Salmonella* contamination in medicinal materials and preparations has attracted widespread attention. The Pharmacopoeia of the People’s Republic of China (ChP) stipulates that *Salmonella* should not be detected per 10 g or 1 mL of herb-free raw powder, oral preparations of traditional Chinese medicines containing raw herb powder, and orally consumed and injection preparations [4]. However, the current method for *Salmonella* detection in ChP involves microbial counting, which is time-consuming and labor-intensive. Although molecular biology methods like polymerase chain reaction (PCR) [5,6] and loop-mediated isothermal amplification (LAMP) [7] have shown effectiveness in reducing detection time and improving sensitivity, they are hindered by complex pre-treatment procedures [8] and the requirement for specialized equipment and skilled technicians for operation [9]. Hence, the development of a simple and rapid microbiological detection method is imperative for ensuring the quality of traditional Chinese medicine.

In recent years, microfluidic biosensors have undergone rapid development, gradually replacing many functions of traditional biochemical laboratories [10,11]. These devices are increasingly being widely developed and applied for rapid microbiological detection [12]. These microfluidic chip devices have minimal volume, with many functions integrated on chips measuring just a few centimeters [13]. The internal dimensions of these chips range from micrometers to millimeters, resulting in sample and reagent consumption in the nanoliter and picoliter range [14]. Additionally, detection is fast and easy to operate. This technology has found extensive use in fields such as food science, biomedicine, pharmacy, and proteomics, leading to significant breakthroughs. In the context of bacterial detection, the identification and separation of target bacteria are crucial. Various separation methods based on magnetic separation [15,16], inertial microfluidics [17,18], acoustics [19], and other technologies have been developed. Among these, magnetic separation is the most commonly used due to its ease of operation and high enrichment rates under the influence of an applied magnetic field [20,21,22]. Due to the complex composition of traditional Chinese medicine and the extremely low content of target bacteria, signal amplification is often necessary through methods such as fluorescence [23,24], chemical [25,26], and enzymatic approaches [27]. Producing color signals via enzyme-catalyzed chromogenic substrates represents a very intuitive form of signal amplification [28,29]. Compared with natural enzymes, nanocatalysts have overcome the shortcomings of easy loss of activity, difficult preservation and high cost, and become a research hotspot [30,31].

**Scheme 1:**
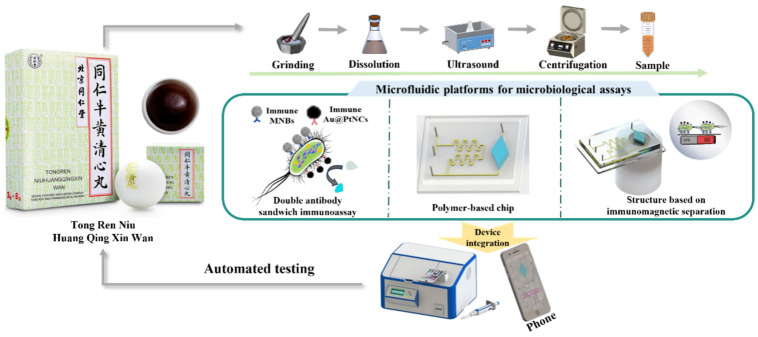
Microfluidic biosensor for quantitative detection of *Salmonella* in traditional Chinese medicine.

Here, a special microfluidic biosensor was proposed, which utilizes gold@platinum nanocatalysts (Au@PtNCs) for signal amplification to achieve rapid, automated, and highly sensitive detection of *Salmonella* in traditional Chinese medicine. Magnetic separation was used to capture *Salmonella* from complex substrate, and Au@PtNCs were used for signal amplification due to their good catalytic activity. As illustrated in Scheme 1, the traditional Chinese medicine preparation was firstly pretreated to obtain the bacterial sample solution according to the ChP. Then, three solutions of immune magnetic nanobeads (MNBs), immune Au@PtNCs, and bacterial samples were mixed up in the proposed microfluidic chip to form a MNBs-*Salmonella*-Au@PtNCs sandwich structure through an antigen–antibody immune reaction. Subsequently, the MNBs-*Salmonella*-Au@PtNCs complex was captured by a magnetic field. Cleaning was performed to remove impurities and excess Au@PtNCs, then hydrogen peroxide-tetramethylbenzidine substrate (H_2_O_2_-TMB) was added to initiate a color reaction. Finally, the bacterial concentration was calculated according to the color. The whole detection process, including mixing, incubation, separation, catalyzing, and detection, was integrated into a portable device, which achieved full process automation. The primary innovation of our study lies in the application of a microfluidic biosensor for the quantitative detection of *Salmonella* in traditional Chinese medicine.

## 2. Materials and Methods

### 2.1. Materials

*Salmonella typhimurium* (ATCC 14082) served as the target bacteria, while *Staphylococcus aureus* (ATCC 25293), *Listeria monocytogenes* (ATCC 13932), *Escherichia coli* O157 (ATCC 43888), *Vibrio parahaemolyticus* (ATCC 17802), *Salmonella enteritidis* (ATCC 13076), and *Vibrio cholerae* (ATCC 14035) were designated as non-target bacteria. Monoclonal antibody (mAb) against *Salmonella* (2.5 mg/mL) and anti-*Salmonella* polyclonal antibody (pAb) (1 mg/mL) were, respectively, procured from Meridian Company (Pennsylvania, USA) and Abcam Company (Cambridge, UK). Spiked samples were prepared using China Beijing Tongrentang Group Lit’s Niu Huang Qing Xin Wan. Propanetriol from Tianjin Damao Chemical Reagent Company was employed for bacterial preservation, while HEPES from Beijing Lamblade Bio-technology Company was used to adjust the pH for antibody coupling to Au@PtNCs. NaH_2_PO_4_·2H_2_O and Na_2_HPO_4_·7H_2_O obtained from Tianjin Guangfu Science and Technology Development Company were utilized for preparing PB (0.1 M) buffer. Sucrose sourced from Tianjin Fuchen Chemical Reagent Company was also utilized. Carboxy magnetic nano-beads (180 nm) purchased from Shanghai Aorun Micro-Nano New Material Technology Company were employed in the synthesis of immune MNBs. Polyvinyl pyrrolidone (PVP, 10 kDa) from Sigma (Saint Louis, MO, USA) and trisodium citrate and ascorbic acid from Aladdin (Shanghai, China) were used to synthesize Au@PtNCs. Bovine serum albumin (BSA) from Shanghai Allrun Nano Science & Technology Co., Ltd. was used for blocking, while 1-(3-dimethylaminopropyl)-3-ethylcarbodiimide (EDC) from Alfa Aesar was used for activating carboxylic acid groups in MNBs. NHS obtained from Sigma-Aldrich (Shanghai, China) was utilized to enhance the efficiency of the coupling reaction by pairing with EDC. The transmission electron microscope used was the FEI Tecnai G2 F30 from FEI, USA, with an accelerating voltage of 300 KV and a point resolution of 0.2 nm.

### 2.2. Preparation of Target Bacteria

First, 5 mL of sterilized nutrient broth was taken, and 100 µL of *Salmonella* species was added. This mixture was incubated at 37 °C on a shaker for 14 h. Then, 100 µL of *Salmonella* diluted 10^4^ times was taken, and 50 µL was spread on an agar medium and incubated at 37 °C for 12 h. After the end of the culture period, the *Salmonella* colonies were allowed to incubate at 37 °C for another 12 h, and the cultured bacterial solution was stored at 4 °C.

### 2.3. Preparation of Immune MNBs

The carboxyl groups on the surface of MNBs were first activated using prepared EDC (10 mg/mL PB 6.0) and NHS (10 mg/mL PB 6.0) at 220 rpm for 1 h at room temperature. After washing and redissolution, 100 µg of *Salmonella* polyclonal antibody was added, and incubated on a shaker at 180 rpm at 37 °C for 2 h. Subsequently, BSA (100 mg/mL PB 7.4) was added to block for 1 h. After washing twice with PBS (0.01 M, pH 7.4), the immune MNBs (1 mg/mL) were redissolved in 1 mL of PBS (0.01 M, pH 7.4) containing 0.25 g sucrose and 10 mg BSA and stored at 4 °C.

### 2.4. Preparation of Au@PtNCs

The Au@PtNCs were pre-synthesized following our previously reported study [32]. Specifically, 31 µL of 13 nm AuNPs nanoparticles (10 nM) and 20 µL of PVP (20 wt%) were added to 969 µL of deionized water and stirred for 5 min. Subsequently, 60 µL of H_2_PtCl_6_ (100 mM) and 40 µL of ascorbic acid (100 mg/mL) were added to the solution. The reaction was carried out at 70 °C for 30 min, during which the color of the solution changed from red to black. After completion, the solution was cooled to room temperature and stored at 4 °C for use (More details could be found from the Supporting Information).

### 2.5. Preparation of Immune Au@PtNCs

A total of 500 µL of Au@PtNCs was centrifuged (5500 rpm, 10 min) and washed with PBS (0.01 M, pH 7.4) to remove excess PVP. Subsequently, 500 µL of PBS (0.01 M, pH 7.4) was used for redissolution, and 50 µL of HEPES (0.1 M) solution and 37.5 µg of mAb against *Salmonella* were added. After incubation for 3 h, the immune Au@PtNCs were blocked by BSA (1 wt%) for 1.5 h. Excess antibody was then removed by centrifugation at 5500 rpm for 10 min at 4 °C. Finally, the immune Au@PtNCs were redissolved in 500 µL PB (0.01 M, pH 8.0) containing sucrose (30 wt%), BSA (3 wt%), and PVP (3 wt%) and stored at 4 °C (More details could be found from the Supporting Information).

### 2.6. Microfluidic Chip Design and Fabrication

**Figure 1:**
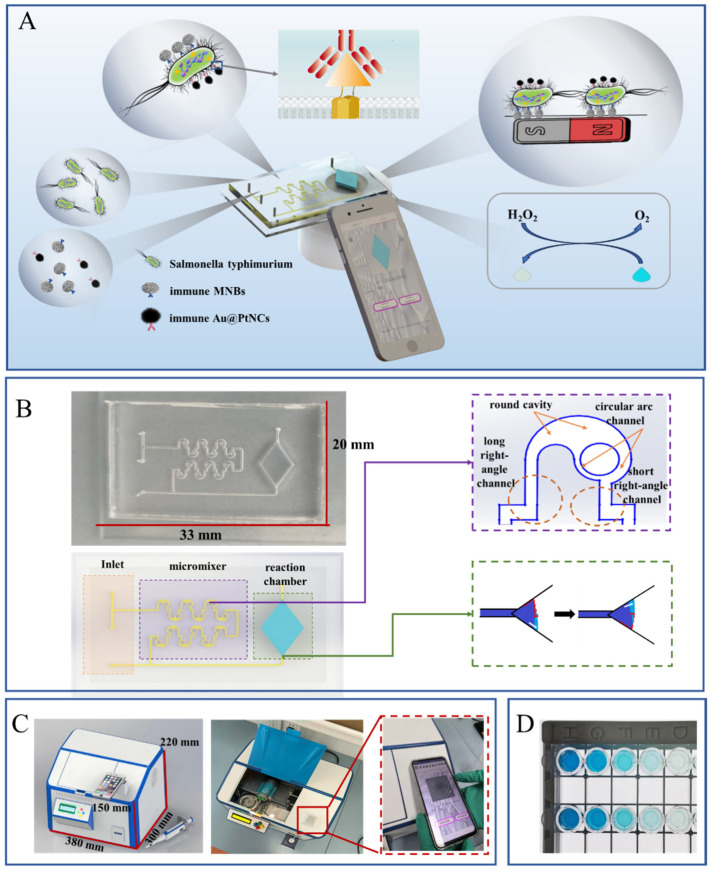
Schematic of the microfluidic biosensor for *Salmonella* detection. (**A**) The principle and the whole procedure of the biosensor. (**B**) Structure of the microfluidic chip. (**C**) The portable device and the smartphone app. (**D**) Color change of the catalysate for different amounts of Au@PtNCs.

The microfluidic chip used was of the size 33 mm × 20 mm × 3.5 mm and mainly comprised an inlet area, micromixer, reaction chamber, and outlet. As depicted in Figure 1B, the inlet area and outlet channel had a width and height of 0.3 mm. The micromixer consisted of 12 mixing units, and each mixing unit was composed of four parts: a long vertical corner channel, a circular cavity, a circular arc channel, and a short vertical corner channel. The circular cavity promoted secondary flow perpendicular to the flow direction, while the arc channel facilitated strong collision at the exit, directing the fluids into the short vertical corner channel. This configuration increased the contact area between the fluids, resulting in a “sudden expansion effect” that enhanced mixing efficiency. In a rhombic cavity, as the cavity filled, the contact angle of the curved liquid surface adhering to one wall increased until a critical value was reached, but since the rhombic cavity did not have circular symmetry, the liquid advanced after reaching the critical value. The liquid in contact with the inner wall advanced faster than the leading edge, and the momentum made it less likely that the edge of the curved liquid surface would adhere to the inner wall again until the contact angle at the edge of the curved liquid surface had increased to the point where it is difficult to advance. The bent liquid surface advanced toward the outlet with a transient delay and subsequently realized the process from adhesion to movement at a position where the average motion of the bent liquid surface was simultaneously relatively uniform on both sides of the chamber. Considering the size of the reaction system, the volume of the reaction chamber was designed to be 90 µL. Previous studies have indicated that a diamond-shaped reaction cell is more effective in avoiding the generation of air bubbles compared to a circular reaction chamber [33].

The molds for the microfluidic chips were designed using SolidWorks software 2020. Photosensitive resin molds were then printed using a Formlabs 3D printer and cured under UV light. The PDMS prepolymers and curing agent, mixed at a mass ratio of 10:1, were stirred and cast into the molds. The cast PDMS channels were dried in an oven at 65 °C for 2 h. After demolding, the PDMS channels underwent plasma treatment for 2 min along with clean glass sheets. Subsequently, the treated PDMS channels and glass sheets were bonded together to make the microfluidic chip complete.

### 2.7. Detection of Target Bacteria

Before bacterial detection, the microfluidic chip underwent several preparatory steps. Initially, the chip was rinsed with 200 µL of alcohol (75%, *v*/*v*) followed by 200 µL of deionized water. After blocking with 200 µL BSA (1%) for 2 h, it was stored at 4 °C for later use.

The detection method of this proposed biosensor is shown in Figure 1A. First, the chip was rinsed with 200 µL of deionized water to remove any residual BSA. Then, the chip was placed in the portable device and the pipeline was connected. Subsequently, the portable device was used to activate the peristaltic pump and simultaneously deliver 100 µL of *Salmonella* typhimurium at a concentration ranging from 9 × 10^0^ to 9 × 10^6^ CFU/mL, along with 8 µL of immune Au@PtNCs solution with 6 µg immune MNBs, into the microfluidic chip. After flowing through the micromixer, these two solutions were fully mixed. Then, the mixture was incubated in the reaction chamber for 50 min to form the MNBs-*Salmonella*-Au@PtNCs complex. Next, the magnetic field was introduced to capture the MNBs-*Salmonella*-Au@PtNCs complex at the bottom of the reaction chamber. To remove excess immune Au@PtNCs and impurities, 100 µL of PBS was injected into the lower inlet of the chip. Following this, 100 µL of H_2_O_2_-TMB was injected from the same inlet and allowed to react with the MNBs-*Salmonella*-Au@PtNCs complex for 24 min. Once the reaction was over, the color was captured by the camera and analyzed using the smartphone app.

### 2.8. Detection of the Target Bacteria in Niu Huang Qing Xin Wan

The test material was prepared following the guidelines outlined in the Pharmacopoeia of the People’s Republic of China [4]. Briefly, 10g of Niu Huang Qing Xin Wan was dissolved in pancreatic casein peptone to prepare a 1:10 solution. Subsequently, the test samples containing 2.6 × 10^2^ to 2.6 × 10^6^ bacteria was prepared by adding 100 µL of bacterial solution to every 900 µL of the prepared solution. The analysis of the test samples was conducted according to the method outlined in the Section 2.7.

### 2.9. Development of the Portable Device

As Figure 1C shows, the portable device was developed using embedded control technology to control various functions such as sonication, solution dispensing, magnetic separation and experiment progress monitoring. A peristaltic pump (T100-S500&WX10-18-H’longer, Baoding, China) precisely and automatically controlled the flow of solutions for sampling, mixing, incubation, washing, and reaction. A multi-way valve (QHF-SV04-X-S-T06-K1.2-S Runze Fluid, Nanjing, China) was controlled by the processor to draw sample solution, color developer, detergent, etc., separately. Ultrasound was integrated on the device to preprocess the sample. A white LED lamp with a diffuser was set below the reaction chamber of the chip to take photos of the colored solution. All components were integrated and controlled via an arduino single-chip microcomputer. An enclosure (size: 380 mm × 220 mm × 340 mm) was 3D printed and painted with white matte paint to create an optical darkroom for housing the device. After the reaction, a sound and light signal prompted the operator to capture the image of the chromogenic reaction product using a smartphone. The smartphone app was then utilized to analyze the data and obtain real-time reaction results. As shown in Figure 1D, the solution catalyzed by different concentrations of Au@PtNCs showed blues with different saturation, so the saturation (S) of the Hue-Saturation-Value color model (HSV) was used to quantify the color of the image, and the bacterial concentration was able to be further calculated through the calibration curve (More details for the camera, software and phone could be found from the Supporting Information).

## 3. Results and Discussion

### 3.1. Characterization of AuNPs and Au@PtNCs

**Figure 2:**
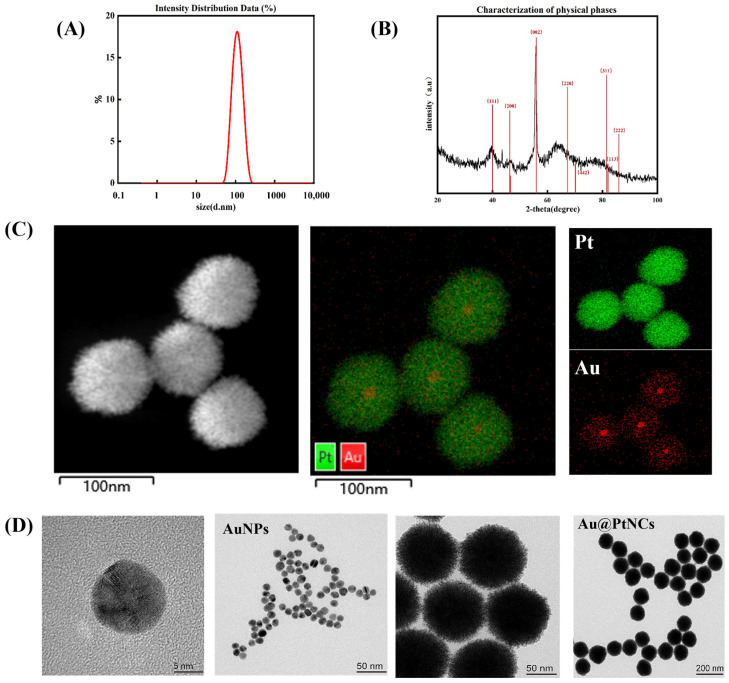
Characterization of materials. (**A**) DLS images of Au@PtNCs. (**B**) XRD pattern of Au@PtNCs. (**C**) EDS elemental mapping of Au@PtNCs. (**D**) TEM images of AuNPs and Au@PtNCs.

As depicted in Figure 2A, through dynamic light scattering (DLS) measurement, the mean diameter of the Au@PtNCs was analyzed to be 106 nm. As depicted in Figure 2B, the peak positions of the X-ray diffraction (XRD) patterns show diffraction peaks at 2θ = 40.04°, 46.28°, 46.53°, 67.31°, 70.18°, 81.50°, 82.01°, and 85.95°, which correspond to the Au and Pt face-centered cubic structures (111), (200), (002), (220), (442), (311), (113), and (222) crystal planes, respectively. The (311), (113), and (222) crystal planes were in full agreement with the experimentally expected results. As depicted in Figure 2C, energy-dispersive X-ray spectroscopy (EDS) elemental mapping further confirmed the exact distribution of Au andPt elements in one particle, with Au as the core and Pt wrapped in the outer layer, forming a core-shell structure. As depicted in Figure 2D, scanning transmission electron microscopy (TEM) was employed to confirm the formation of desired nanostructures such as AuNPs and Au@PtNCs. TEM examination clearly revealed that all the particles were fairly uniform in size and shape with well-defined mesopores.

### 3.2. Verification of the Effectiveness of Microfluidic Chip

The presence of air bubbles in the reaction chamber can significantly impact the accuracy of HSV values obtained through photos taken with the smartphone app, leading to potential bias in the final determination of bacterial concentration.

**Figure 3:**
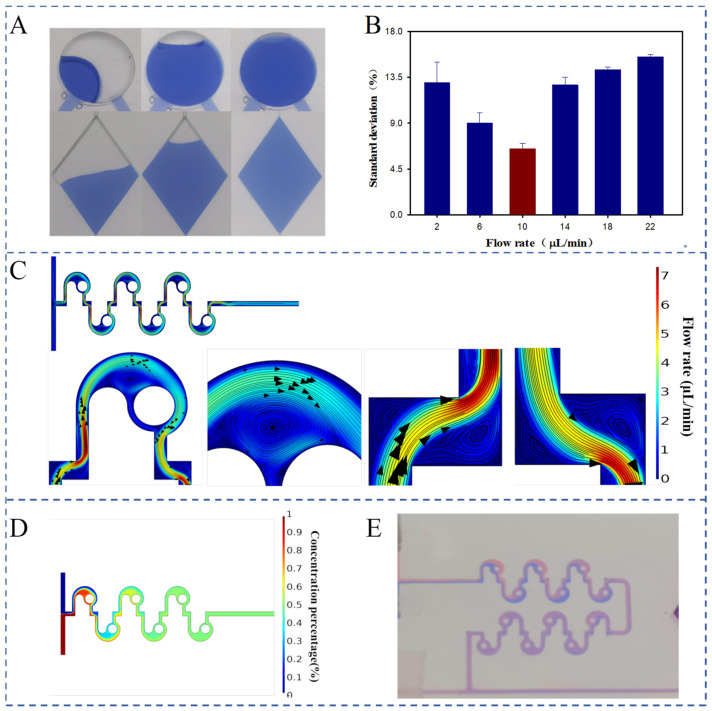
Validation of the performance of microfluidic chip. (**A**) Comparison round chambers with diamond-shaped chambers. (**B**) Comparison of flow rate effects (N = 3). (**C**) Simulation of the micro-mixing zone. (**D**) Simulated concentration schematic of the concentration in the micro-mixing zone. (**E**) Ink mixing experiment.

Bubbles in the reaction chamber will significantly affect the reaction process and subsequent image analysis, so the chamber shape needed to be optimized to avoid bubbles. Ink experiments show that compared with a circular cavity, a diamond cavity can effectively avoid the generation of bubbles. As shown in Figure 3A, during the filling of the circular chamber with ink, due to the high symmetry of the circular chamber, it is easy to generate bubbles. Once the inlet and outlet are connected, these bubbles cannot be discharged. In contrast, the rhombic chamber lacks the symmetrical properties of a circle. When the solution enters the rhombus chamber, the liquid adsorbed on one side of the V-shaped chamber will continue to accumulate, making the contact angle between the liquid and the edge increase. When the contact angle reaches a critical value, the subsequent liquid entering the chamber will tend to accumulate from the other side of the V-shaped chamber. Finally, the air is completely discharged at the outlet, enhancing the reliability of the reaction and the accuracy of the final determination of bacterial concentration.

The flow rate in the channel is one of the most important factors affecting the mixing effect of the micro-mixing zone. In order to verify the actual effect of different flow rates, blue ink and red ink were transported into the micro-mixing zone together. The mixed solution was photographed using a camera at different flow rates. ImageJ was used to analyze the color images, and the mixing uniformity of ink was evaluated according to the standard deviation of the R (RED) value in RGB. Figure 3B shows that the standard deviation (SD) decreased and then increased as the flow rate increased from 2 µL/min to 22 µL/min. At a flow rate of 10 µL/min, the SD decreased to a minimum of 6.5, indicating that the mixing efficiency reached the highest value. Therefore, 10 µL/min was chosen as the optimal flow rate for this microfluidic biosensor.

Further, COMSOL Multiphysics was used to simulate the mixing process in the micro-mixing zone. The streamline diagram in Figure 3C visually shows the secondary flow generated in the micro-mixing zone, which significantly enhanced the mixing effect. Additionally, the concentration diagram shown in Figure 3D illustrates that two different liquids were uniformly mixed within the sixth mixing unit, indicating an improved mixing effect. As shown in Figure 3E, the simulation results were confirmed by the experimental findings.

### 3.3. Verification of the Performance of Magnetic Fields

**Figure 4:**
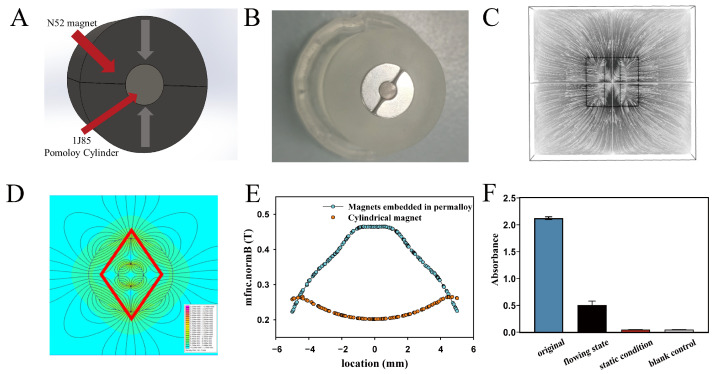
Performance of magnetic field. (**A**) The structure of the magnetic field. (**B**) Photo of the magnetic field. (**C**) Magnetic induction line of the magnetic field. (**D**) The strength of the magnetic field and the location of the reaction chamber. (**E**) Comparison of the magnetic field density of this magnetic field with that of a circular magnet. (**F**) Magnetic recovery results for this magnetic field (N = 3).

The magnetic field plays a crucial role in capturing the magnetic nano-beads (MNBs), thereby directly impacting the accuracy of measured bacterial concentration. In order to further improve the capture efficiency of the biosensor, a high-gradient magnetic field was creatively designed. As shown in Figure 4A,B, the high-gradient magnetic field consisted of two horseshoe-shaped N52 magnets with an outer diameter of 4.5 mm and an inner diameter of 0.5 mm wrapped around a permalloy cylinder with a radius of 0.5 mm and a length of 10 mm. The two horseshoe magnets were radially magnetized and mutually exclusive. Simulations with COMSOL Multiphysics were used to evaluate the effectiveness of the designed magnetic field. As shown in Figure 4C, the density of magnetic inductance lines increased significantly from the outer edge of the magnet inward, indicating that the magnetic field gradient in this region was large. The high magnetic field strength and gradient effectively enhance dthe magnetic force on the MNBs in the solution, thus realizing the high enrichment rate of the MNBs.

Magnetic field intensity simulation experiments were conducted using FEMM 4.2. As Figure 4D shows, the location of the high intensity of the magnetic field was point distributed and located inside the reaction chamber, which was utilized to make the MNBs to gather in a point manner. Magnetic field simulation experiments were performed using COMSOL Multiphysics to obtain the magnetic field density at 1 mm. As shown in Figure 4E, compared with cylindrical magnets, the improved combined magnetic field had a greater magnetic field strength and gradient, providing a greater magnetic force. The magnetic force on a single MNB in a magnetic field can be expressed as [34]:

(1) $$F_{m}=x\mu_{0}\upsilon_{p}H\nabla H,$$

where x is the difference between the volume magnetic susceptibility of the particle and the surrounding fluid in which it is immersed; $\mu_{0}$ is the permeability of free space, which is a constant (4π × 10^−7^ N/A^2^); $\upsilon_{p}$ denotes the volume of the MNBs; and H and ∇H denote the strength and gradient of the magnetic field, respectively. The MNBs used in the study had a diameter of 180 nm and a volumetric magnetization of about 350 emu/cm^3^, and since the volume magnetization of the fluid was much smaller than that of the MNBs, the volume magnetization of the MNBs was be approximated as the difference between the volume magnetization of the MNBs and the fluid. The magnetic field density *B* is related to the magnetization strength and the applied magnetic field strength *H* by the following equation:

(2) $$H=\frac{B}{\mu_{0}}-M,$$

where M is the magnetization, and $\mu_{0}$ is a physical quantity that describes the state of magnetisation of a magnetic medium. M in this Equation (2) can be expressed as follows:

(3) M=xH,

Substituting Equations (2) and (3) into Equation (1) yields the magnetic force acting on the MNBs as:

(4) $$F_{m}=x\mu_{0}\upsilon_{p}\frac{B\nabla B}{\mu_{0}\left(1+x\right)},$$

Thus, the force of the magnetic field on the MNBs designed in this paper is set as $F_{m1}$, and the same size cylindrical magnet is set as $F_{m2}$, which can be obtained as follows:

(5) $$\frac{F_{m1}}{F_{m2}}=\frac{B_{1}{\nabla B}_{1}}{B_{2}{\nabla B}_{2}}$$

Therefore, substituting the COMSOL Multiphysics simulation data into Equation (5) to calculate, it can be obtained that the magnetic force of the improved magnetic field on the MNBs is 6.3 times that of the conventional magnetic field, indicating that the improved magnetic field has better magnetic capture effect.

Further, the capture efficiency of the magnetic field was verified by experiments. A total of 200 µL of MNBs solution (150 µg/mL) was passed into the microfluidic chip at a flow rate of 10 µL/min, and the designed magnetic field was set below the detection cavity to capture the MNBs. There was a good linear relationship between the concentration of the MNBs solution and the absorbance value of the solution, so MNBs content in the initial MNBs solution and the solution discharged from the chip were compared by absorbance. The effects of two capture forms, i.e., capture in the flowing state and at a static condition (capture time 5 min), were compared. As shown in Figure 4F, in flowing state, only a few (23%) escaped MNBs were detected in the waste liquid, especially in the static state; almost no escaped MNBs were detected in the waste liquid, indicating that the magnetic field had good capture efficiency.

### 3.4. Optimization of Microfluidic Biosensor

**Figure 5:**
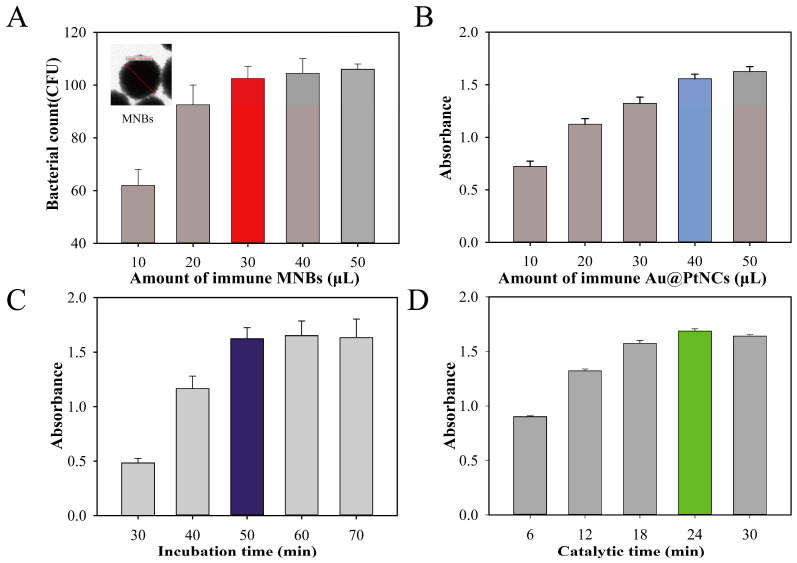
Optimization of microfluidic biosensor (N = 3). (**A**) Optimization of the amount of immune MNBs. (**B**) Optimization of the amount of immune Au@PtNCs. (**C**) Optimization of the incubation time. (**D**) Optimization of the catalytic time.

In this microfluidic biosensor, the factors affecting the detection sensitivity mainly include the amount of immune MNBs and immune Au@PtNCs, the incubation time and the catalytic time. Firstly, the amount of immune MNBs was optimized. Briefly, different amounts of immune MNBs were added to 500 µL of bacterial solution with a concentration of 2.6 × 10^6^ CFU/mL, mixed and incubated for 50 min, separated and cleaned by magnetic separation, and then the captured bacteria were counted by culture method after dilution. As shown in Figure 5A, the amount of immune MNBs increased from 10 µg to 30 µg, the number of colonies increased from 62 to 102 CFU, and there was no significant change when the amount of immune MNBs was increased to 50 µg. Thus, the optimal amount of 30 µg for the MNBs was used.

To optimize the amount of immune Au@PtNCs, 500 µL of *Salmonella*-MNBs complex with a concentration of 2.6 × 10^6^ CFU/mL and different amounts of immune Au@PtNCs were mixed to form a *Salmonella*-MNBs-Au@PtNCs complex. Then, 100 µL of the complex solution was taken to catalyze the reaction of 100 µL of H_2_O_2_-TMB for 20 min, and the absorbance of the reactants was measured. As shown in Figure 5B, the absorbance increased from 0.72 to 1.56 as the amount of immune Au@PtNCs changed from 10 to 40 µL and it did not increase significantly when the amount of immune Au@PtNCs further changed. Therefore, the optimal volume of 40 µL for immune Au@PtNCs was used.

Furthermore, 500 µL of 2.6 × 10^6^ CFU/mL of *Salmonella* was mixed with 40 µL of immune Au@PtNCs and 30 µg of immune MNBs. Then, the mixture was incubated at room temperature for different time spans, ranging from 30 min to 70 min. A total of 100 µL of the mixture was taken to catalyze the reaction of 100 µL of H_2_O_2_-TMB for 20 min and the absorbance of the reactants was measured. From Figure 5C, it can be seen that the incubation time increased from 30 to 50 min and further increased to 60 min with no significant change in absorbance. Therefore, the optimal incubation time was found to be 50 min.

To investigate the catalytic timing of H_2_O_2_-TMB, 500 µL of 1.2 × 10^6^ CFU/mL of *Salmonella* was mixed with 30 µL of immune Au@PtNCs and 20 µL of immune MNBs. After 50 min of incubation, 100 µL of the mixture was taken to catalyze the reaction of 100 µL of H_2_O_2_-TMB over different time spans. As shown in Figure 5D, the absorbance significantly increased as the catalytic time changed from 6 min to 24 min, and did not lead to a significant increase when the catalytic time further changed to 30 min. This indicates that 24 min was sufficient for catalysis.

### 3.5. Performance Evaluation of This Proposed Biosensor

**Figure 6:**
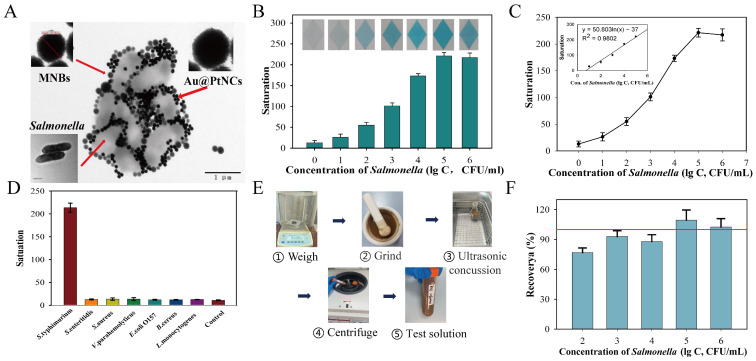
Performance of microfluidic biosensor. (**A**) TEM image of MNBs-*Salmonella*-Au@PtNCs complex. (**B**) Saturation for *Salmonella* at different concentrations (N = 3). (**C**) Calibration curve of the biosensor (N = 3). (**D**) Specificity of the biosensor. (**E**) Pre-treatment process of the Niu Huang Qing Xin Wan (N = 3). (**F**) Recovery rate of spiked *Salmonella* typhimurium samples (N = 3).

As shown in Figure 6A, TEM confirmed the formation of the MNBs-*Salmonella*-Au@PtNCs complex. To determine the bacterial concentration of an unknown sample, experiments were conducted under optimal conditions to establish the biosensor’s calibration curve. According to the method outlined in Section 2.7, different concentrations of *Salmonella* from 9 × 10^0^ to 9 × 10^6^ CFU/mL were detected by this proposed biosensor, and the smartphone app was used to calculate the bacterial concentration according to the saturation value. As shown in Figure 5C and Figure 6B, when the bacterial concentration increased from 9 × 10^1^ CFU/mL to 9 × 10^5^ CFU/mL, the saturation increased from 26.37 to 221.25. A good linear relationship was obtained, and could be expressed by *y* = 50.803 ln(*x*) − 37 (R^2^ = 0.98). When the concentration of the bacterial solution increased to 9 × 10^6^ CFU/mL, the absorbance decreased, which may have been influenced by the hook effect. The limit of detection (LoD) for the proposed biosensor was determined as the lowest point of the linear range, which was 90 CFU/mL, meeting the requirements for bacterial detection in traditional Chinese medicine. As shown in Table 1, the proposed biosensor exhibited significant advantages in both detection time and sensitivity. Notably, this biosensor was able automate the entire detection process.

To verify the specificity of this microfluidic biosensor, 6.2 × 10^6^ CFU/mL of the target bacteria and six other bacteria were detected. As can be seen from Figure 6D, the saturation of the target bacteria was significantly higher than that of the other bacteria, indicating that the biosensor had good specificity. Notably, the biosensor exhibited the capability to differentiate between different serotypes of *Salmonella*, further enhancing its utility and reliability.

To further evaluate the feasibility of the biosensor in the detection of real Chinese medicine samples, the spiked Niu Huang Qing Xin Wan samples with different concentrations of *Salmonella* were detected using this biosensor. As shown in Figure 6E, the pre-treatment process was carried out according to the process specified in the ChP. The viable bacterial counting method was used to determine the concentration of the bacteria at the same time. As shown in Figure 6F, the average recoveries of the spiked samples ranged from 76.8% to 109.5% with standard deviations of 4.7% to 10.1%. The deviation between the test results and the viable bacterial counting method may be attributed to (1) the influence of viscous excipients such as honey in the Niu Huang Qing Xin Wan samples on the immune response, and (2) the fact that some components in the Niu Huang Qing Xin Wan affected the pH of the solution, making it deviate from the isoelectric point of the antibody.

## 4. Conclusions

This study presents a novel approach to detecting *Salmonella* in traditional Chinese medicine using a microfluidic biosensor enhanced by Au@PtNCs signal amplification and a magnetic field. The biosensor automates the entire detection process, including mixing, incubation, separation, and catalyzing, enabling rapid detection of *Salmonella* at concentrations as low as 90 CFU/mL. Notably, the biosensor offers advantages such as high specificity, sensitivity, low cost, and automation. Compared with the traditional culture method, this proposed biosensor shortens the detection time from 3–7 d to just 74 min, making it a promising tool for microbiological detection in traditional Chinese medicine. It is worth mentioning that the total cost for this microfluidic biosensor does not exceed US $2.6, and all the components listed in supporting information. This study provides a breakthrough new technological method for microbiological detection in traditional Chinese medicine, which has broad development prospects. However, this study was limited to the detection of live bacteria and the area of differentiation and quantitative detection of live and dead bacteria needs to be addressed by subsequent researchers. Moreover, the equipment developed in this study needs to be used with a smartphone, pending integration studies by subsequent researchers.

## Figures and Tables

**Table 1:** Comparison of this proposed biosensor with some recently reported methods for bacterial detection.

Methods	Target	LOD (CFU/mL)	Linear Range (CFU/mL)	Total Time (h)	References
Culture	*C. auris*	1.3 × 10^2^	——	48	[35]
LFIA	*S. typhimurium*	10 × 10^2^	10 × 10^2^–1.0 × 10^7^	0.5	[36]
Fluorescent	*S. typhimurium*	1.0 × 10^2^	1.0 × 10^2^–10 × 10^7^	0.7	[37]
Electrochemical	*S. typhimurium*	0.94 × 10^2^	1.3 × 10^2^–13 × 10^7^	0.8	[38]
PCR	*E. Coli* O157:H7	1.0 × 10^2^	0.1 × 10^2^–0.01 × 10^7^	1.3	[39]
Colorimetric	*S. typhimurium*	56 × 10^2^	100 × 10^2^–0.1 × 10^7^	0.8	[40]
Colorimetric	*S. typhimurium*	0.90 × 10^2^	0.9 × 10^2^–0.09 × 10^7^	1.2	This biosensor

## Data Availability

Data are contained within the article.

## Supplementary Materials

The following supporting information can be downloaded at: https://www.mdpi.com/article/10.3390/bios15010010/s1.
