# Supplementary material for: A Microfluidic Biosensor for Quantitative Detection of Salmonella in Traditional Chinese Medicine
Source: Biosensors (Basel). 2024 Dec 27;15(1):10. doi: 10.3390/bios15010010 (PMC11763935; doi:10.3390/bios15010010)
Supplement: Supplementary file 1 [file biosensors-15-00010-s001.zip › biosensors-3385988-supplementary.pdf]

A microfluidic biosensor for quantitative detection of Salmo-nella in Traditional Chinese Medicine

More details for the camera, software and phone.

The said camera is the camera that comes with the cell phone; the said software is the self-developed quantitative analysis of Chinese medicine microorganisms V1.0, logging in the system must be configured for the software platform: operating system: Android system, the software environment: Eclipse+JDK+Android SDK+ADT, Android Studio+Android SDK, JDK+Android SDK, ADT-Bundle. ADT-Bundle; the specific configuration of the said cell phone is as follows: CPU: recommended but not limited to Snapdragon 8+Gen 1, Snapdragon 8Gen 2, Tenguiz 9000, Tenguiz 9200, Kirin 9000; Memory: this system storage occupies 7.4MB, the memory requirements are low, it is recommended to use 4G or larger memory; cell phone pixels: 13 million or more; resolution: recommended 1080P or above.

Testing Cost Estimator

Testing Cost Estimator

Disposables	Norm	Quantities	price of item	total price(¥)
Microfluidic chip	g	4	0.7	2.8
Immune MNBs	μL	20	0.13	2.7
Immune Au@PtNCs	μL	30	0.3	9
Antibodies	μL	10	0.4	4
				18.5

Preparation of immune MNBs

Take 1 mg of Orun Magnetic Beads to 3 mL of PB (pH 6.0, 0.01 M), wash with shaking for 1 min, followed by magnetic recovery for 10 min, remove the supernatant, repeat the wash 3 times, and redissolve to 3 mL of PB (pH 6.0, 0.01 M). Dissolve 10 mg of EDC and 10 mg of NHSS in 1 mL of PB 6.0 and 100 μL of each was added to the solution in rapid sequence and reacted on a shaker at 37°C, 220 rpm for 1 h. Magnetic recovery was carried out for 10 min and washed twice with PB 6.0, the last time without re-dissolution. 100 μg of salmadolizumab was added to 3 mL of PB (pH 8.4, 0.01 M) and subsequently precipitated using its re-solubilized Orun magnetic beads, which were ultrasonically shaken to ensure dispersion of the beads, and the reaction was carried out on a shaker at 37°C, 220 rpm, for 2 h. Forty mg of skimmed milk was dissolved in 400 μL of PB (pH 7.4, 0.01 M), which was added to the above reaction solution, and the reaction was carried out for 1 h. At the end of this time, the reaction was magnetically recovered for 10 min and washed twice with PBS (pH 7.4, 0.01 M), and the precipitate was redissolved in 1 mL of a special complex solution.

Preparation of Au@PtNCs

Soak brand-new glass vials and brand-new rotors overnight using aqua regia, after which the vials and rotors are cleaned (tap water rinse, ultrapure water rinse, magnetic stirrer spin clean; to clean the rotors, place them in a 15 mL centrifuge tube and vortex to oscillate; when removing the rotors, place the rotors on clean disposable plastic gloves). The glycerol in one magnetic stirrer was warmed up to 70 °C in advance. The above clean glass vial with the rotor was placed on another magnetic stirrer.969 μL of ultrapure water was added and adjusted to the appropriate rotation speed, followed

by the rapid addition of 31 μL of AuNPs nanoparticles and 20 μL of PVP, and stirred uniformly for 5 min.60 μL of H2PtCl6 and 40 μL of Ascorbic acid, mixed uniformly, transferred to an oil bath magnetic stirrer, adjusted to the appropriate speed, and reacted for 30 min until the color of the solution changed from red to black. Cooled to room temperature and stored at 4°C. To remove the excess PVP, centrifuge the solution at 4°C (3000 g, 10 min) and wash once with PBS.

Preparation of immune Au@PtNCs

Take 500 μL of synthesized platinum pellet solution, 4°C, centrifuge for 10 min, wash once using PBS (0.01 M, pH=7.4) to remove excess PVP. Wash the rotor and glass vials, place the rotor in a 15 mL centrifuge tube using ultrapure water, then place the rotor in a glass vial with ultrapure water and place on a magnetic stirrer,1000 rpm, 5 min, and the rotor was cleaned 3 times. After the platinum pellet is cleaned, it is re-dissolved with 500 μL of PBS (0.01 M, pH=7.4), 50 μL of HEPES solution (0.1 M) is added and 37.5 μg of antibody is added (at about 500 rpm), and electrostatic adsorption is performed for 3.5 h. The redundant sites are sealed by the addition of 500 μL of BSA solution (2%) for a period of 1.5 h. The platinum pellet is washed with ultrapure water in a glass vial. At 4°C, centrifugation (5500 rpm, 10 min) and wash once with PBS (0.01 M, pH=7.4) to remove excess antibody. Dissolve with 500 μL of specialized reagent solution (10% sucrose + 1% skim milk + 1% PVP, configured as 30% sucrose + 3% skim milk + 3% PVP in equal volumes, in PB (0.01 M, pH 8.0)) and store at 4°C.
